# Pan-Cancer Analysis Predicts the Immunological and Prognostic Role of ZC3H12C in KIRC

**DOI:** 10.1155/2022/4541571

**Published:** 2022-06-26

**Authors:** Yanping Yue, Yumeng Wu, Dakun Zhao, Biao Wu, Xuming Wu, Jibin Liu, Lei Yang, Aiguo Shen

**Affiliations:** ^1^Nantong University, Nantong, Jiangsu, China; ^2^Cancer Research Center Nantong, Affiliated Tumor Hospital of Nantong University, Nantong, Jiangsu, China; ^3^Nantong Fourth People's Hospital, Nantong, Jiangsu, China; ^4^Deparment of Oncology, Affiliated Tumor Hospital of Nantong University, Nantong, Jiangsu, China

## Abstract

ZC3H12C is an important member of the CCCH-zinc finger protein family and is mainly involved in host immune and inflammatory diseases. However, its abnormal expression and prognostic value in cancer have not yet been established. Through comparative analysis of the Cancer Genome Atlas (TCGA) database, we found that ZC3H12C is the most relevant to the prognosis, grade, and stage of renal clear cell carcinoma (ccRCC) across 33 cancers. With the help of patient transcription and clinical data from the TCGA and GEO (GSE53757, GSE36895) databases, we determined that in the immune environment of patients with ccRCC, ZC3H12C was clearly negatively correlated with Tregs and was significantly positively correlated with monocytes. In addition, protein phosphorylation and DNA methylation analysis also showed that ZC3H12C negatively regulates the role of cancer in ccRCC. Our research may provide new insights into ccRCC immunotherapy and bring forth novel biomarkers and therapeutic targets.

## 1. Introduction

There are about 400,000 annual cases of renal cell carcinoma (RCC) worldwide, making it one of the common malignancies of the urinary system [1]. As a subtype of kidney cancer, clear cell renal cell carcinoma (ccRCC, KIRC) has the highest mortality. There is a lack of satisfactory treatment options for ccRCC. Immunotherapies have revolutionized cancer treatment in recent years, with immune checkpoint blockade (ICB) being particularly important. As an outstandingly heterogeneous disease, especially in immunotherapy, valuable biomarkers which help to individualize treatment options are still lacking for RCC [2]. Several studies have identified CD8+ T cells, Tregs, and macrophages as risk factors for RCC [3]. In addition, some studies have shown that the proportion of CD8+ T cells, Tregs, and M0 macrophages increases with the increase of Fuhrman grade, while the proportion of dendritic cells, mast cells, monocytes, and resting CD4+ T memory decreases [4–6]. These data suggest that tumor-infiltrating immune cells (TIICs) are important determinants of prognosis in ccRCC. In addition, the development and improvement of public databases provide an opportunity to discover new immunotherapeutic targets via big data analysis [7].

ZC3H12C, located on chromosome 11q22.3, is an RNA binding protein (RBP). It is also a CCCH-zinc finger protein family member mainly involved in host immune and inflammatory diseases, such as psoriasis [8]. Among this family of proteins, the expression of ZC3H12C is highly specific in macrophages [9]. A previous study showed that ZC3H12C enhances the negative regulation of Gfi1 through nuclear factor *κ*B (NF-*κ*B) activation during inflammation in macrophages. NF-*κ*B is a transcriptional factor that regulates a battery of genes that are critical to innate and adaptive immunity, as well as tumor progression. Other studies have demonstrated that microRNA 21 (miR-21) is a positive regulator of the NF-*κ*B pathway [10]. Moreover, ZC3H12C is regulated by the transcription of interferon (IFN) signals, and it inhibits tumor necrosis factor *α-*(TNF*α*-) induced chemokines to regulate vascular endothelial inflammation [11]. Some studies have also shown that ZC3H12C is negatively correlated with lung adenocarcinoma and colorectal cancer [12–14]. However, no one has yet studied whether ZC3H12C affects ccRCC prognosis.

In this study, ZC3H12C has been studied using bioinformatic tools such as TIMER2.0, GEPIA, and LinkedOmics among others. We found that the ZC3H12C gene is underexpressed across several cancer types and associated with survival. We focused on the correlation between differences in ZC3H12C transcription and protein expression in ccRCC patients. Our data show that ZC3H12C is precisely regulated by phosphorylation and methylation in ccRCC, thereby affecting other proteins or RNA. Through its role in tumor immunity, ZC3H12C plays a prognostic role in ccRCC. This study helps us to understand the role of ZC3H12C in ccRCC immunotherapy.

## 2. Materials and Methods

### 2.1. Patients and Data Files

Patients with 33 different tumors in the TCGA cohort and patients with KIRC (kidney renal clear cell carcinoma), in the GEO dataset (GSE53757 and GSE36895), were studied. Tumor mRNA expression data were obtained from http://xena.ucsc.edu/ and https://www.ncbi.nlm.nih.gov/. Four pairs of precious ccRCC tissues were provided by Nantong Cancer Hospital.

### 2.2. Differential Gene Expression Analysis

Using TIMER2.0 (http://timer.cistrome.org/) and data from the TCGA project to analyze the expression of ZC3H12C in different tumors and adjacent normal tissues, some tumors with abnormal or low ZC3H12C expression in normal tissue were obtained by the GEPIA 2 (http://gepia2.cancer-pku.cn) using the “Expression DIY” command. The UALCAN portal (http://ualcan.path.uab.edu/) was used to analyze oncology data, enabling us to assess the mRNA expression of the TCGA datasets and protein expression analysis on the CPTAC (Consortium for Clinical Proteome Analysis of Tumor) dataset. The starBase (http://www.sysu.edu.cn/), a comprehensive online tool, allowed us to use largescale mRNA data from the TCGA datasets to analyze gene expression in 32 different cancers. In this way, we analyzed various functions of ZC3H12C with multiomics data in LinkedOmics (http://www.linkedomics.org/).

### 2.3. Significant Survival Prognosis Analysis

The Kaplan–Meier plotter (http://kmplot.com/analysis/), LinkedOmics, and starBase were used to obtain an overall survival (OS) significance map data of ZC3H12C across all TCGA tumors. The “Clinical” module provided by the TISIDB tool (http://cis.hku.hk/TISIDB/) was used to measure the relationship between ZC3H12C mRNA levels and the OS of human cancers, cancer stages, and tumor grades. We also selected the corresponding database in LinkedOmics to assess the relationship between ZC3H12C, pathological staging, and TNM staging in KIRC. We performed univariate and multivariate Cox regression to build the nomogram model. The forest map generated a *P* value, HR, and 95% CI of each variable with the help of different tools (http://sangerbox.com/ and https://www.aclbi.com/static/index.html#/).

### 2.4. Comprehensive Protein Phosphorylation and DNA Methylation Analysis

In UALCAN, we separately explored the expression levels of total protein or phosphoprotein (phosphorylated at S123) of ZC3H12C (NP_203748.1) between the primary tumor and normal tissues in KIRC. We evaluated the methylation level of ZC3H12C by searching the “Methylation” under “TCGA analysis” at the same time. Next, SurvivalMeth (http://bio-igdata.hrbmu.edu.cn/survivalmeth/) illustrated the role of ZC3H12C promoter methylation in KIRC prognoses. Next, we used MEXPRESS to observe changes in the methylation of CpG sites in KIRC under different ZC3H12C expression levels. LinkedOmics enabled us to visualize genes associated with ZC3H12C methylation in KIRC.

### 2.5. Systematical Immune Infiltration Analysis

BioGPS (http://biogps.org/), a free, extensible, and customizable gene annotation portal, was used as a complete resource for understanding gene and protein function. We used this dataset to visualize the expression levels of ZC3H12C mRNA in human tissues. Following that, we compared these results to the ZC3H12C expression in immune cells (i.e., CD8+ T cells, Tregs, and monocytes) using the TIMER2.0 web server and GEO merged data across KIRC. The CIBERSORT algorithm was applied to estimate immune infiltration. To calculate *P* values and partial correlation (cor) values, we used Pearson's rank correlation, and the data were visualized as a scatter plot. Subsequently, the ZC3H12C expression was also compared to immune subtypes within the TISIDB across a wide variety of human cancers. Moreover, KIRC immune subtypes were studied in detail.

### 2.6. Related Gene Enrichment Analysis

We searched cBioPortal (http://www.cbioportal.org/) using “Kidney” and “Kidney Renal Clear Cell Carcinoma” (TCGA, PanCancer Atlas) search terms. Subsequently, we identified a series of genes for KEGG (Kyoto Encyclopedia of Genes and Genomes) pathway analysis. Next, a functional annotation chart was generated using DAVID (Database for Annotation, Visualization, and Integrated Discovery) with the combination of the selected identifiers (“OFFICIAL_GENE_SYMBOL”) and species (“Homo sapiens”) (https://david.ncifcrf.gov/). The enriched pathways were finally visualized with the data visualization package “ggplot2”in *R*. In the following step, we used RNAct (https://rnact.crg.eu/) in a similar fashion using “ZC3H12C”as the query. Using the intersection of the genes in these two databases, we obtained 50 genes displayed in a Venn gram. Based on immunology research, we decided to screen three of these genes. The correlation was then calculated using TIMER2.0.

### 2.7. Western Blot

Next, protein was loaded onto a 10% sodium dodecyl-sulfate polyacrylamide gel electrophoresis (SDS-PAGE) gel for electrophoresis. Protein bands were then transferred to polyvinylidene difluoride (PVDF) membranes. After blocking nonspecific binding using 5% (w/v) skimmed milk, the membranes were incubated overnight at 4°C with primary antibody against ZC3H12C (1 : 1000, rabbit, GeneTex, Texas, USA) and GAPDH (1 : 5000, mouse, Abcam, Cambridge, UK).

### 2.8. Statistical Analysis

**Figure 1:**
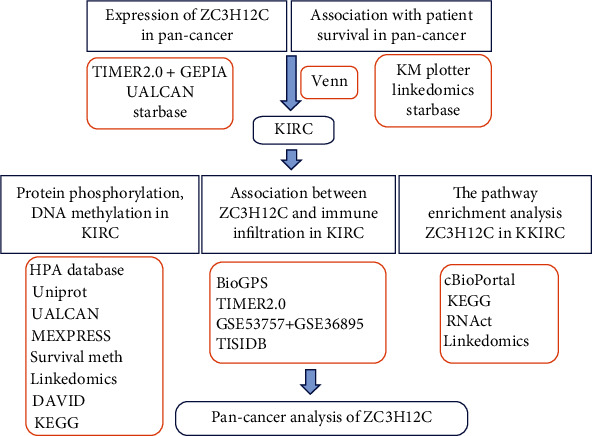
The roadmap of the research.

All statistical analyses were performed in *R* version 3.6.2. The Wilcoxon signed-rank test was used to study the function of ZC3H12C in clinical pathologic features. In addition, the Kaplan–Meier method and Cox regression were used to evaluate the role of the ZC3H12C expression in KIRC prognosis. For Cox regression analysis, we incorporated the variables with *P* < 0.1 in univariate Cox regression into multivariate Cox regression. A bilateral *P* value <0.05 was considered statistically significant. This method flow is shown in Figure 1.

## 3. Results

### 3.1. ZC3H12C Is Universally Downregulated in Different Human Cancers

**Figure 2:**
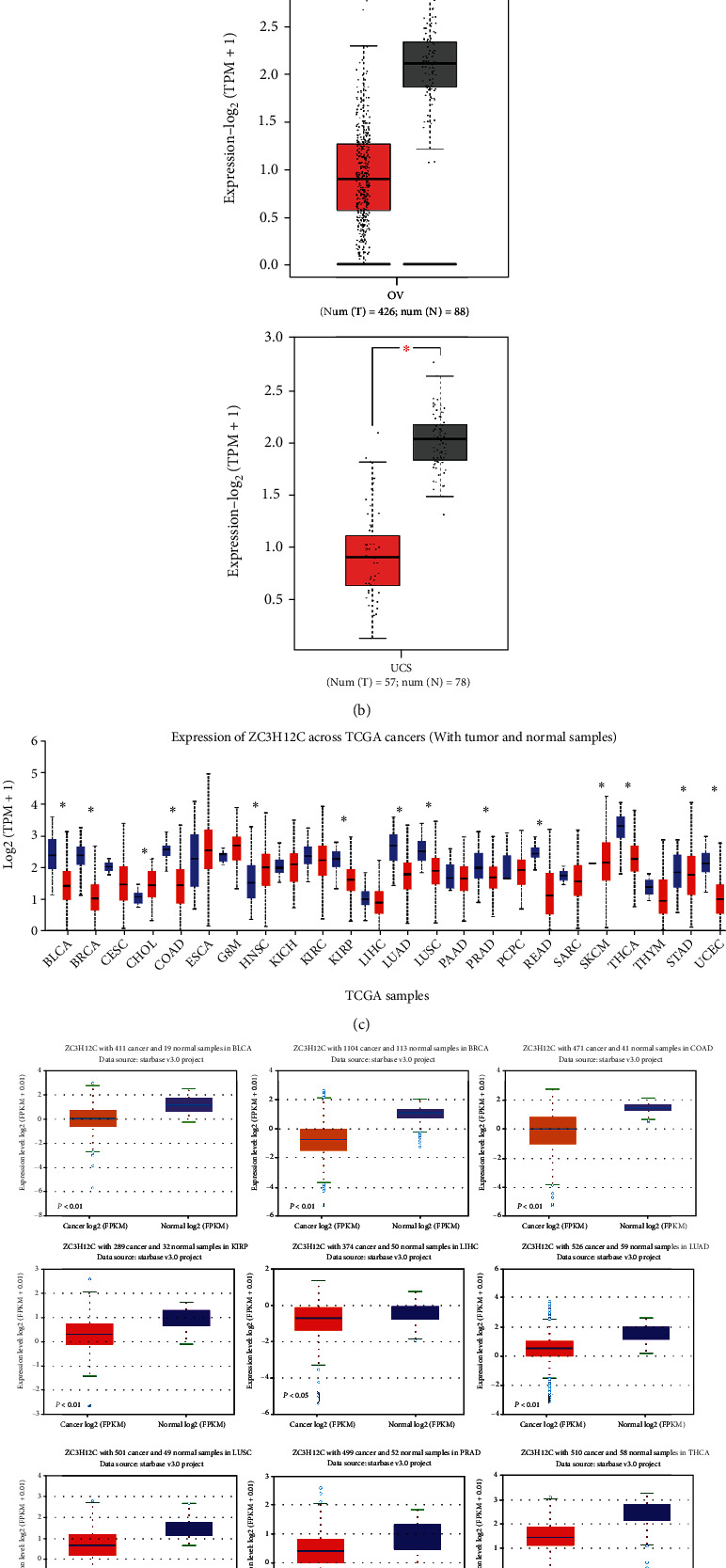
Differential expression of the ZC3H12C gene in different tumor types. (a) The ZC3H12C gene expression was analyzed using TIMER2.0 in various cancer types. ^∗^*P* < 0.05, ^∗∗^*P* < 0.01, ^∗∗∗^*P* < 0.001. (b) DLBC, GBM, LGG, SKCM, TGCT, and THYM were included in the TCGA project, and the GTEx database was used as a control. Data are presented as box plots. ^∗^*P* < 0.05. (c, d) The ZC3H12C total mRNA expression level in human cancers and normal tissues was also analyzed using data from the TCGA dataset in different platforms, such as the UALCAN and the starBase. ^∗^*P* < 0.05.

The differential expression of ZC3H12C in different cancers is likely due to its corresponding biological functions. First, we analyzed the expression of ZC3H12C in different cancer types in the TCGA database by searching for TIMER2.0. We found that the ZC3H12C expression was significantly higher in adjacent tissues compared to diseased tissues in BLCA (bladder urothelial carcinoma), BRCA (breast invasive carcinoma), COAD (colon adenocarcinoma), KIRC, KIRP (kidney renal papillary cell carcinoma), LUAD (lung adenocarcinoma), LUSC (lung squamous cell carcinoma), PRAD (prostate adenocarcinoma), READ (rectum adenocarcinoma), THCA (thyroid carcinoma), and UCEC (uterine corpus endometrial carcinoma) (Figure 2(a)). In addition, using normal tissues from the GTEx data as controls, we further confirmed with GEPIA that ZC3H12C was significantly lower in OV (ovarian serous cystadenocarcinoma) and UCS (uterine carcinosarcoma) (Figure 2(b)). In addition, the ZC3H12C expression was significantly lower in ACC (adrenocortical carcinoma), PRAD, SARC (sarcoma), and SKCM (skin cutaneous melanoma). Moreover, UALCAN analysis showed that ZC3H12C was significantly downregulated in 10 tumors, namely, BLCA, BRCA, COAD, KIRP, LUSC, LUAD, PRAD, READ, THCA, and UCEC (Figure 2(c)). In addition, using the starBase database, we found that the expression of ZC3H12C in BLCA, BRCA, COAD, KIRP, LIHC, LUAD, LUSC, PRAD, THCA, and UCEC was lower compared to paracancerous tissues (Figure 2(d)). Thus, ZC3H12C is downregulated in almost all cancers.

### 3.2. ZC3H12C Expression Is Closely Associated with Patient Survival in Different Human Cancers

**Figure 3:**
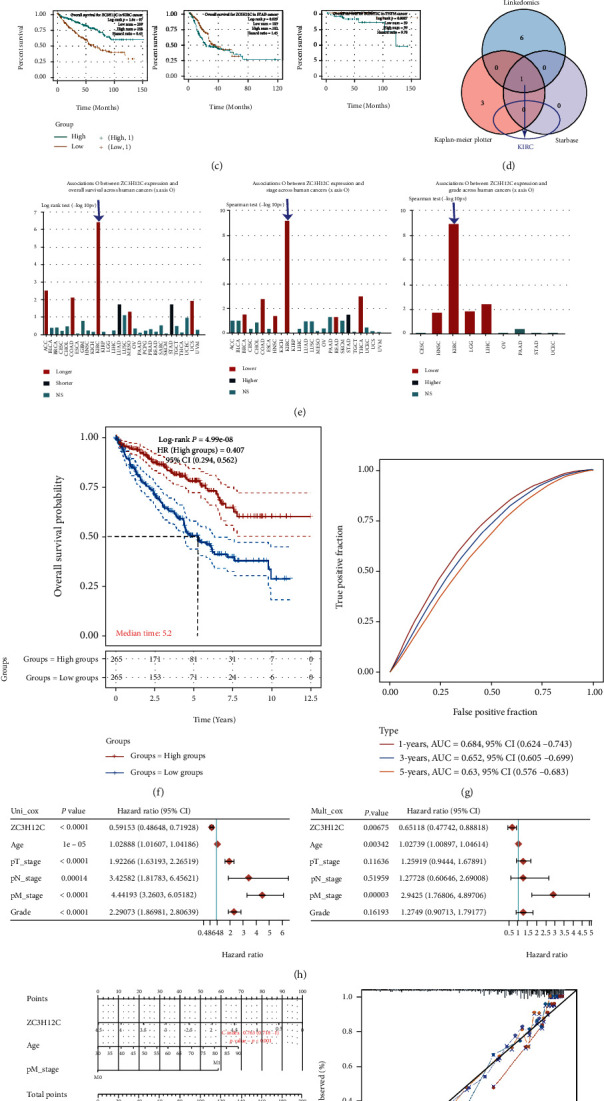
ZC3H12C gene expression is correlated with survival rates in the TCGA cancer cohort. (a)–(c) A survival map and Kaplan–Meier curves with positive results presented for the different tumors in the TCGA based on the ZC3H12C gene expression. The Kaplan–Meier plot and LinkedOmics data are also shown. (d) Comparative analysis of data in (a)–(c) was shown as Venn diagrams. (e) ZC3H12C is closely related to longer overall survival, lower stage, and lower grade across human cancers. (f, g) The survival curve and AUC analysis of the model are displayed. (h) Forest map illustrates the effect of multivariate and univariate Cox proportional hazard models on the ZC3H12C expression in KIRC. (i) A nomogram was used to predict the 1-, 2-, and 3-year overall survival of KIRC cancer patients. Calibration curve for the overall survival nomogram model. The blue line, red line, and orange line represent the observed 1-, 2-, and 3-year deaths, respectively. (j) Pathological stage, TNM stage, and ZC3H12C are closely related in KIRC. (k) The results of western blot of four paired tissues are shown.

Based on the TCGA data set, we analyzed the ZC3H12C expression in different types of cancer and found a correlation between its expression and the prognoses for different patients. First, the high ZC3H12C expression is associated with favorable outcomes in ESCA (esophageal carcinoma), KIRC, and PAAD (pancreatic adenocarcinoma), while the opposite is true in LUAD, STAD (stomach adenocarcinoma), THYM (thymoma), and UCEC (Figure 3(a)). Based on analysis of the LinkedOmics database, we found that the high ZC3H12C expression is significantly associated with good outcomes in ACC, COADREAD (colon adenocarcinoma/rectum adenocarcinoma esophageal carcinoma), GBMLGG (glioma), KIRC, KIPAN (pan-kidney cohort (KIRC + KICH + KIRP)), MESO (mesothelioma), and UCS (Figure 3(b)). The same was true for STAD and STES (stomach and esophageal carcinoma) but not for THYM in which the high ZC3H12C expression is significantly associated with poor prognosis. According to the starBase database, the upregulated ZC3H12C expression is linked to favorable prognoses for KIRC, while the opposite is true in STAD and THYM (Figure 3(c)). Second, through the intersection of these three databases, we found that the ZC3H12C expression is implicated in prognoses of patients with KIRC (Figure 3(d)). Therefore, we decided to focus on analyzing the importance of ZC3H12C in KIRC. Importantly, this point was supported by TISIDB data (Figure 3(e)). Compared to other cancers, in KIRC, high level of ZC3H12C is most significantly associated with longer OS (Figure 3(f)). Results from area under the curve (AUC) analysis prove the high accuracy of this model (Figure 3(g)). In addition, although the results in the ICGC data are not ideal, this also suggests that further research is needed (Figure S1).

Clinical data and ZC3H12C expression data were downloaded from the TCGA database. Univariate analysis showed that ZC3H12C expression, age, T stage, *N* stage, M stage, and tumor grade are all related to patient prognosis, while multivariate analysis revealed that ZC3H12C expression, age, and M stage are linked to patient survival (Figure 3(h)). Moreover, the Cox regression algorithm was used to establish a related prognostic model while histograms and forest plots were used for data visualization (Figure 3(i)). KIRC prognosis is highly correlated to the ZC3H12C expression with *C* index of 0.763 for this model. Further, using the LinkedOmics database, we found that the ZC3H12C expression was significantly associated with pathological stage, T stage, M stage, and *N* stage (Figure 3(j)). Finally, immunoblotting of four pairs of RCC tissues and adjacent tissues confirm upregulation of the ZC3H12C protein expression in adjacent tissues (Figure 3(k)). In conclusion, the ZC3H12C expression is closely associated with patient survival in different human cancers, and this is the most obvious in KIRC.

### 3.3. Association between ZC3H12C Expression and Protein Phosphorylation and DNA Methylation in KIRC

**Figure 4:**
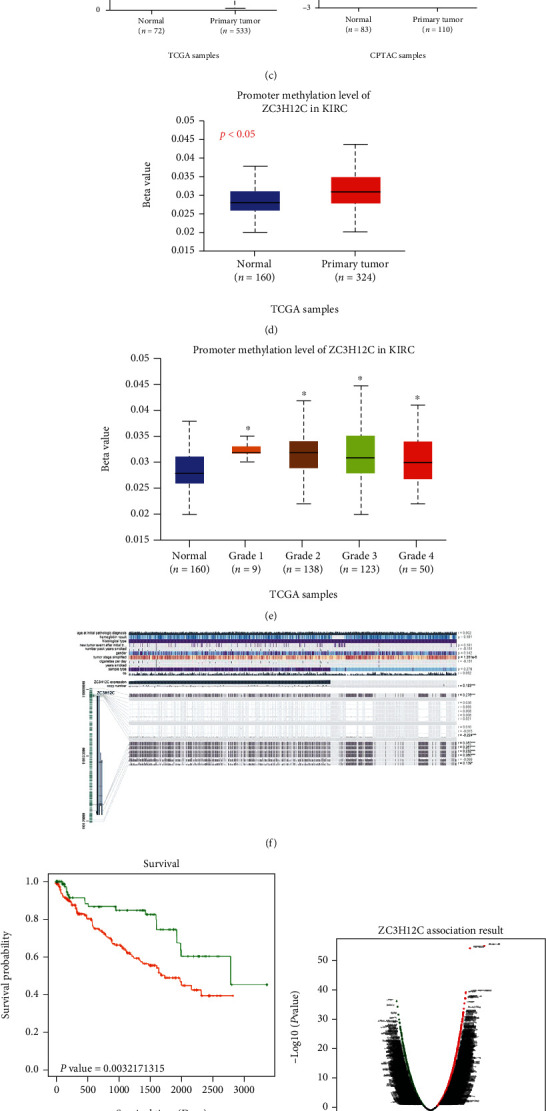
Analysis of ZC3H12C phosphorylation and DNA methylation in various tumors. (a, b) All detected proteins are shown along with the sites of protein phosphorylation. (c) We examined the expression of the ZC3H12C phosphoprotein (S123 site) using UALCAN on the CPTAC dataset. The ZC3H12C protein diagram shows the phosphoprotein sites that had positive results. (d, e) For methylation of ZC3H12C and its association with grade, we supply a box plot in KIRC. (f) ZC3H12C is implicated in methylation mutations at 7 CpG sites in KIRC. (g, i) ZC3H12C methylation-related genes and pathway enrichment bubble diagram are displayed.

Using the HPA database, we found the upregulated expression of ZC3H12C renal tissues (Figure 4(a)). We used the CPTAC dataset to compare the differences in ZC3H12C phosphorylation levels in paracancerous and primary tumor tissues in KIRC. The KIRC phosphorylation site has been published in UniProt (Figures 4(b) and 4(c)). UALCAN was used to show the phosphorylation levels of the S123 locus in KIRC. We found that the total protein concentration in KIRC was almost similar in cancer and paracancer, while phosphorylated protein levels varied considerably. Research by Iwasaki et al. showed that in response to external stimuli that utilize MyD88 signaling, the ZC3H12A protein is phosphorylated by I*κ*B kinases (IKKs), followed by ubiquitin-dependent degradation. As another member of the same family with similar structure and function, ZC3H12C may also be degraded during phosphorylation.

Using the UALCAN database, we found that methylation of the ZC3H12C promoter increased in tumor tissues and was significantly linked to tumor grade (Figures 3(d) and 3(e)). In the MEXPRESS database, we also found that ZC3H12C is associated with methylation levels at many DNA methylation sites (Figure 3(f)). Next, we used SurvivalMeth to determine the correlation between low ZC3H12C promoter methylation levels and good KIRC prognosis (Figure 3(g)). Importantly, abnormal methylation in the promoter region often leads to inactivation of certain tumor suppressor genes [15]. For example, methylation of the CRB3 [16], Hugl-2 [17] gene promoter region inhibits gene transcription in ccRCC.

Using LinkedOmics to analyze related genes in the methylation database and, subsequently, DAVID to enrich a series of related genes for pathways, we found that these methylation-related genes are implicated in KEGG pathways such as metabolism, cancer, and endocytosis (Figures 3(h) and 3(i)).

### 3.4. Association between ZC3H12C Expression and Immune Infiltration in KIRC

Tumor-infiltrating cells may exhibit pro- or antitumor effects depending on the type of cancer. CD8+ T cells, Tregs, and macrophages are associated with poor prognosis in RCC. Several studies have shown that CD8+ T cells, Tregs, and macrophages increase in abundance as Fuhrman's grade increases, whereas dendritic cells, mast cells, and CD4+ memory T cells decrease. Therefore, we mainly analyzed the correlation between ZC3H12C and these immune cells.

**Figure 5:**
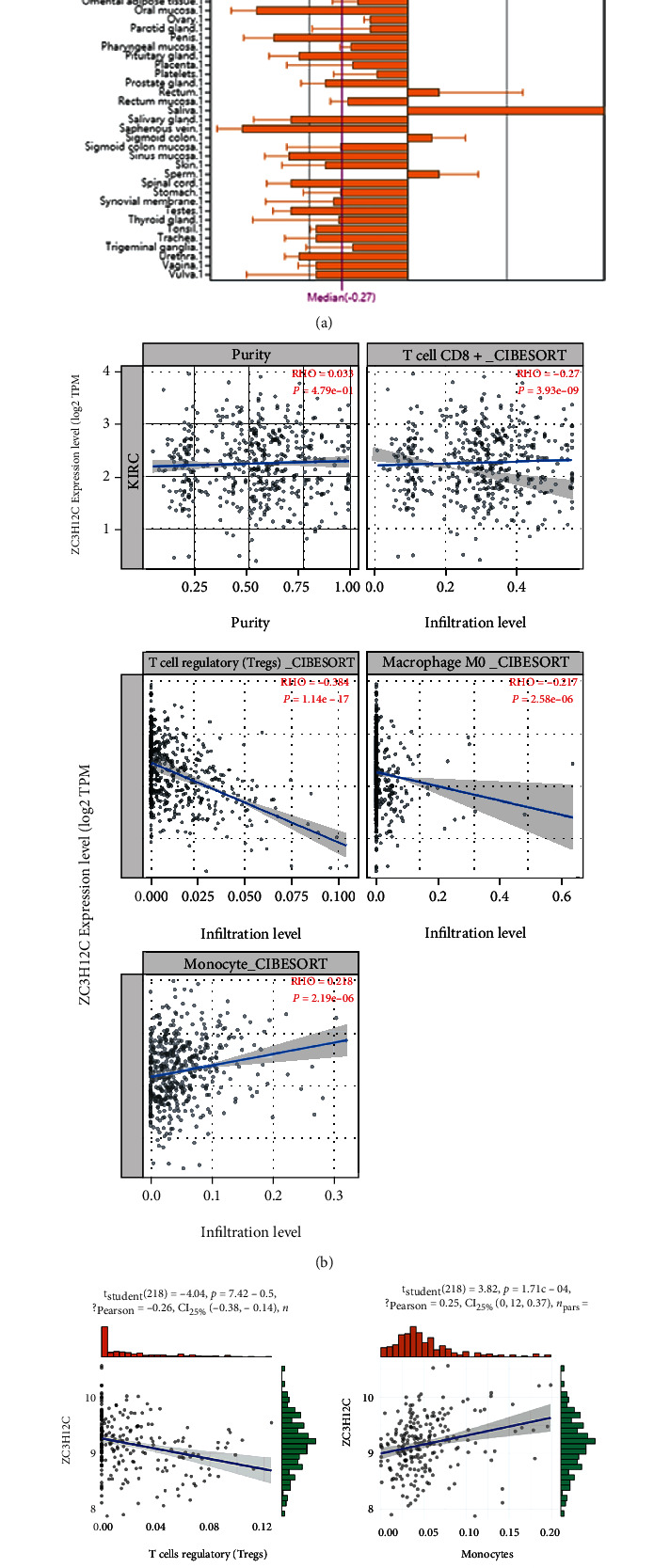
ZC3H12C expression and immune infiltration are correlated. (a) Expression profile for ZC3H12C in human tissues displayed using BioGPS. (b, c) CIBERSORT was used to explore the potential correlation between ZC3H12C gene expression and infiltration of Tregs, monocytes, and CD8 + T cells in the TCGA and GEO datasets. (d, e) The association between ZC3H12C expression and immune subtypes across human cancers is displayed, especially in KIRC subtypes (C1 (wound healing), C2 (IFN-gamma dominant), C3 (inflammatory), C4 (lymphocyte depleted), C5 (immunologically quiet), C6 (TGF-b dominant)).

Using TCGA data and combined GEO data (GSE53757 + GSE36895), we analyzed the relationship between ZC3H12C and various immune cells, respectively. Using BioGPS, we found that ZC3H12C is expressed at very low levels in normal immune cells, such as memory B cells, primary B cells, macrophages, CD8 + T cells, Tregs, and neutrophils (Figure 5(a)). TIMER2.0 calculations showed that ZC3H12C was negatively correlated with CD8+ T cells, Tregs, and M0 macrophages and positively correlated with monocytes using CIBERSORT (Figure 5(b)).

We then combined the matched primary and adjacent normal tissues in the GEO dataset GSE53757 (*n* = 76) and GSE36895 (*n* = 144) and used the CIBERSORT algorithm to calculate the proportion of each immune cells in the KIRC tissue (Table 1). Our data indicates that Tregs and monocytes show consistent significance in the combined GEO data set (Figure 5(c)). As a monocyte chemotactic protein, ZC3H12C holds a positive correlation with monocytes, which proves that our results are reliable. Furthermore, the negative correlation with macrophages shows that ZC3H12C regulates macrophage activation [18, 19]. This shows that ZC3H12C is negatively correlated with Tregs, which may modulate immune homeostasis via a negative regulatory effect in KIRC. In addition, the TISIDB database also confirmed that ZC3H12C is strongly correlated with an immune subtype of KIRC, with the highest expression in the immunologically quiet C5 subtype (Figures 5(d) and 5(e)).

### 3.5. The Pathway Enrichment Analysis of ZC3H12C in KIRC

**Figure 6:**
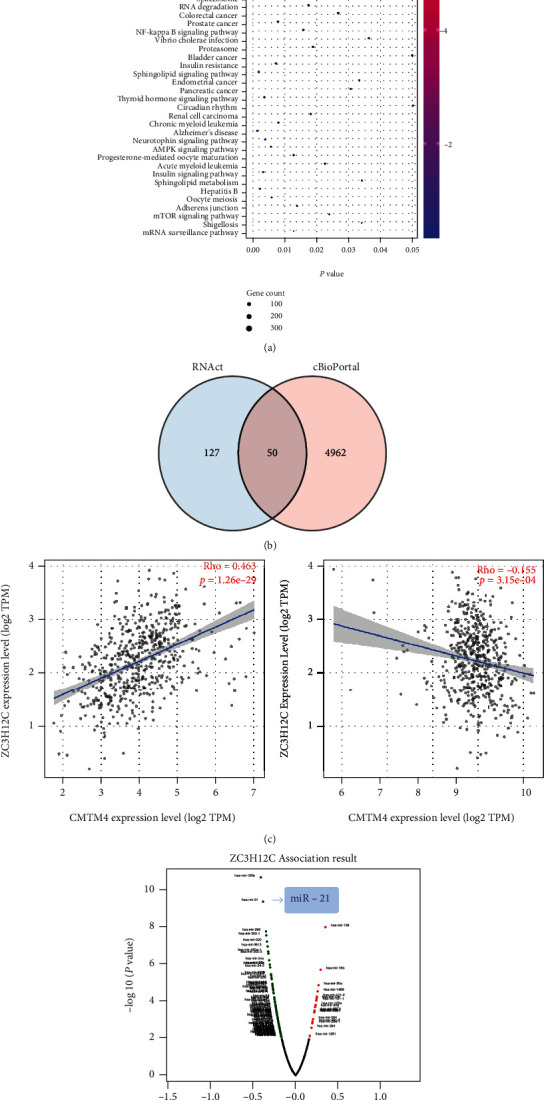
ZC3H12C-related gene enrichment analysis. (a) The available determined ZC3H12C-binding mRNAs using the RNAct tool were obtained. Based on the ZC3H12C-binding and interacted genes, KEGG pathway analysis was performed. (b) Using the LinkedOmics approach, we also obtained the ZC3H12C-correlated microRNAs in TCGA.

We then examined the mechanisms by which ZC3H12C affects ccRCC using genes enriched in the cBioPortal database to perform KEGG enrichment analysis. KEGG data shows that in tumors, ZC3H12C is implicated in RNA transport, cell cycle, RNA degradation, NF-kappa B signaling, RCC, and mTOR signaling. Based on the function of other members of the protein family, we speculate that ZC3H12C may regulate stability of specific mRNAs through its RNase domain. Next, we isolated ZC3H12C target RNA from the RNAct database and cross the coexpressed genes in cBioPortal to obtain the top 50 interacting genes. We identified CMTM4 and CD151 as genes that had been more extensively researched regarding immunity (Figure 6(b)), and their relevance to ZC3H12C is shown in Figure 6(c). LinkedOmics was used to predict ZC3H12C target miRNAs, which were negatively correlated with miR-21 (Figure 6(d)). Previous studies have identified 59 significant immuno-miRNAs markedly related to OS of ccRCC patients including miR-21 [20, 21]. ZC3H12C may have a strong impact on immunity through these molecules.

## 4. Discussion

Studies have shown that ZC3H12C may be involved in the occurrence and progression of human cancers. However, its role in other types of cancers has not been determined. In this study, we conducted comprehensive bioinformatic analyses on the expression and prognostic value of ZC3H12C in 33 cancer types based on the TCGA analysis database. This study showed the downregulation and clinical significance of ZC3H12C in different human cancers and, for the first time, we proved its antitumor effect in KIRC.

As shown in Figure 1, using TCGA data, previous studies into prognostic-related genes in lung adenocarcinoma and colorectal cancer have shown that ZC3H12C is significantly downregulated in these cancers and, thus, implicated in prognosis, suggesting a potential role for ZC3H12C as a potential diagnostic biomarker in these cancers. In this study, for the first time through pan-cancer analysis, we found that ZC3H12C was significantly downregulated in human cancers, especially in 13 cancers. The predicted results of lung adenocarcinoma and colorectal cancer are similar to previous studies. Interestingly, the expression of ZC3H12C in CHOL and HNSC is significantly higher than that of adjacent cancers. We speculate that ZC3H12C may have other complex mechanisms in these two cancers. Our survival analysis using TCGA data showed that in ESCA, KIRC, PAAD, ACC, COADREAD, GBMLGG, KIPAN, MESO, and UCS ZC3H12C is downregulated and associated with improved patient prognosis. The opposite is true in LUAD, STAD, THYM, UCEC, and STES. The specific role of ZC3H12C in each cancer may have a lot to do with the heterogeneity of the cancer itself, and further in-depth experimental verification is needed. However, we are confident that ZC3H12C is protective against KIRC.

We also explored the relationship between ZC3H12C protein phosphorylation and promoter methylation and KIRC. We found that ZC3H12C methylation can be used as a prognostic biomarker in cancer patients. Importantly, we found that although phosphorylated ZC3H12C is highly expressed in cancer, there are no significant differences in total protein between cancer and adjacent cancer. Taken together, we speculate that ZC3H12C is also degraded by phosphorylation.

Studies have shown that ZC3H12C negatively regulates the immune control of psoriasis by inhibiting NF-*κ*B signaling and proinflammatory gene expression in endothelial cells. In addition, ZC3H12C has been identified as a negative regulator of macrophage activation and may be implicated in host immune and inflammatory diseases. Using the TCGA database, we found that ZC3H12C is significantly negatively correlated with CD8+ T cells, Tregs, memory B cells, and macrophages, while it is significantly positively correlated with native B cells, monocytes, and neutrophils. We also merged the GEO dataset for verification and found that ZC3H12C is generally negatively correlated with Tregs and positively correlated with monocytes. It is worthy thinking of ZC3H12C as a monocyte chemoattractant protein inducing protein; so, the above results are not contradictory to the positive correlation of monocytes. Multiple studies have shown that the high Treg expression is implicated in poor prognosis in KIRC. In this work, we show that ZC3H12C may influence prognosis by negatively regulating immune infiltration of tumor cells in KIRC by affecting Tregs. These data provide a new therapeutic target for development of novel immunosuppressive agents.

According to pathway enrichment analysis, ZC3H12C-related genes are significantly implicated in RNA transport, cell cycle, RNA degradation, NF-kappa B signaling pathway, RCC, and mTOR signaling. In addition, in terms of immunity, it is significantly associated with CMTM4, CD151, and miR21. A study has previously shown the high CMTM4 expression in type I RCC (69%) with a statistically significant interaction between CD274 and CMTM6, thereby implicating CMTM4/6 as a potential therapeutic target for type I RCC patients who are resistant to ICB. Another study showed that six of them, including a CD151-derived peptide, were significantly overrepresented in metastases. Experimental data suggest that miR-21 suppresses antitumor T cell-mediated immunity [22]. We speculate that ZC3H12C is regulated by miR-21, and that its regulation affects downstream molecules, including CMTM4 and CD151, thus exerting an inhibitory effect in the immune environment in KIRC.

In conclusion, in our first pan-cancer analysis, we show that ZC3H12C is comprehensively downregulated in tumor tissues, and we reveal its potential functions in clinical prognosis. Our findings indicate that ZC3H12C can be used as an independent prognostic marker for many tumors, with its expression level conferring different prognostic results. Nonetheless, further research is required to unravel the specific role of ZC3H12C in each cancer. Among these cancers, we selected the most relevant, KIRC, for a separate analysis and, for the first time, revealed the relationship between its protein phosphorylation and DNA methylation in KIRC. In addition, the ZC3H12C expression is implicated in immune cell infiltration in KIRC. These findings help to clarify the role of ZC3H12C in tumorigenesis, and they suggest a role as a potential therapeutic target for the development of more precise and personalized immunotherapies in the future.

## 5. Conclusions

Extensive studies have shown that the tumor suppressor molecule ZC3H12C is associated with prognosis, methylation, phosphorylation, and immune invasion of KIRC.

## Figures and Tables

**Table 1:** Correlation of ZC3H12C with TIICs in TCGA and combined GEO set.

Cell type	TCGA	GEO
	*P* val/cor	*P* val/cor
T cell CD8+	^∗∗∗^/-0.27	-0.11
T cell CD4+ naive	-0.022	0.08
T cell CD4+ memory activated	-0.031	-0.12
T cell CD4+ memory resting	0.15	0.08
Tregs	^∗∗∗^/-0.384	^∗∗∗^/-0.26
B cell memory	^∗∗∗^/-0.229	0.02
B cell naive	^∗∗∗^/0.298	^∗^/-0.16
B cell plasma	0.009	-0.13
Monocyte	^∗∗∗^/0.218	^∗∗∗^/0.25
Macrophage M0	^∗∗∗^/-0.217	-0.1
Macrophage M1	^∗∗∗/^0.146	0.09
Macrophage M2	^∗∗^/0.167	0.03
Myeloid dendritic cell activated	0.027	^∗^/-0.14
Myeloid dendritic cell resting	0.053	0.07
Mast cell activated	0.08	-0.04
Mast cell resting	0.093	^∗∗^/0.22
Eosinophil	0.092	0.07
Neutrophil	^∗∗∗^/0.161	0

^*^ means p less than 0.05, ^**^ means p less than 0.01, ^***^ means p less than 0.001.

## Data Availability

The data sets analyzed during the current study are available in the TCGA (https://portal.gdc.cancer.gov/) and GEO repository (https://www.ncbi.nlm.nih.gov/geo/).

## Supplementary Materials

Supplementary Materials Figure S1: the results of ZC3H12C in KIRC with the analysis of the data in ICGC.
