# Supplementary figures and images for: Pan-Cancer Analysis Predicts the Immunological and Prognostic Role of ZC3H12C in KIRC
Source: Biomed Res Int. 2022 Jun 26;2022:4541571. doi: 10.1155/2022/4541571 (PMC9251093; doi:10.1155/2022/4541571)

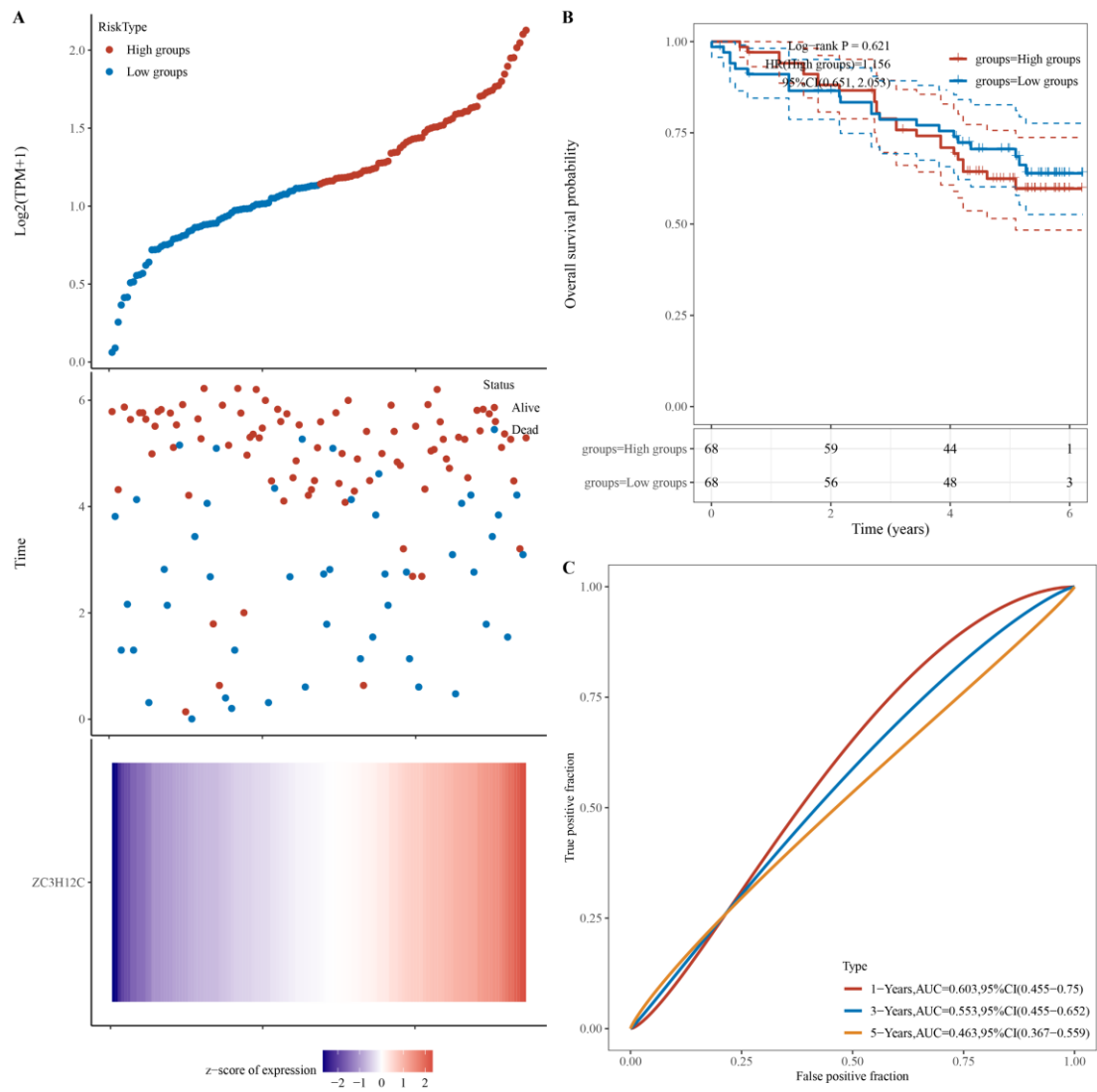

Supplement: Supplementary Materials — Figure S1: the results of ZC3H12C in KIRC with the analysis of the data in ICGC. [file 4541571.f1.pdf]
